# Effect of CPAP therapy on vitamin D status in patients with obstructive sleep apnea and chronic obstructive pulmonary disease overlap syndrome

**DOI:** 10.1007/s11325-025-03324-0

**Published:** 2025-04-17

**Authors:** Kostas Archontogeorgis, Athanasios Voulgaris, Konstantina Chadia, Konstantinos Bonelis, Paschalis Steiropoulos

**Affiliations:** 1 Department of Pneumonology, Medical School, Democritus University of Thrace, Alexandroupolis, 68100 Greece; 2 MSc Program in Sleep Medicine, Medical School, Democritus University of Thrace, Alexandroupolis, Greece

**Keywords:** Chronic obstructive pulmonary disease, Obstructive sleep apnea, Overlap syndrome, Continuous positive airway pressure, Vitamin D

## Abstract

**Purpose:**

Vitamin D (Vit D) deficiency has been associated with both obstructive sleep apnea (OSA) and chronic obstructive pulmonary disease (COPD) as well as with their combination, known as overlap syndrome (OS). There is evidence that continuous positive airway pressure (CPAP) may lead to an increase of Vit D levels in OSA patients. However, the effect of CPAP treatment on Vit D levels in OS patients has yet to be determined. The aim of the present study was to investigate the effect of one year of CPAP therapy on Vit D levels in patients with OS.

**Methods:**

Vit D serum levels were measured in consecutive OS patients at baseline and after one year of CPAP therapy. Compliance with CPAP therapy was assessed by the data retrieved from the CPAP device.

**Results:**

Vit D serum levels were measured in 46 OS patients (43 males). Among participants, 27 had good and 19 poor compliance with CPAP therapy. Results showed that serum Vit D levels increased after 12 months of CPAP therapy from 21.3 ± 8.4 to 23.8 ± 8.7 ng/ml (*p* = 0.001). Moreover, patients with good CPAP compliance demonstrated higher serum 25(OH)D levels compared to those with poor compliance (25.8 ± 7.6 versus 20.4 ± 9.6 ng/ml, respectively; *p* = 0.038).

**Conclusions:**

In conclusion, 12 months of CPAP therapy improved Vit D serum levels in OS patients, more so in compliant patients.

## Introduction

Overlap syndrome (OS) is defined as the concomitant presence of apneas and hypopneas during sleep along with chronic obstructive pulmonary disease (COPD) in the same patient [1]. Although it is unclear whether each disorder increases the prevalence of the other, greater COPD severity is linked to a higher occurrence of obstructive sleep apnea (OSA) [2]. OS affects approximately 1% of the population, has a worse prognosis, and imposes a greater disease burden than either underlying disorder alone [3].

Sympathetic dysregulation, an enhanced pro-inflammatory state, imbalance between oxidant and antioxidant capacity, and endothelial dysfunction are some of the pathophysiological pathways shared between COPD and OSA, all of which can contribute to cardiovascular and other comorbidities [4]. Patients with OS exhibit more pronounced sleep hypoxia than those with either disease alone and are more likely to present daytime symptoms than patients with OSA alone [4, 5]. Currently, continuous positive airway pressure (CPAP) is the recommended treatment for patients with OS, as it improves sleep disordered breathing (apneas, hypopneas and nocturnal oxygenation) and daytime symptoms, regardless of COPD severity [5]. Current data strongly suggest that adherence to CPAP therapy is essential in OSA management and improvement of OSA-related symptoms and complications [6].

Vitamin D (Vit D) is a fat-soluble vitamin essential for bone mineralization, as it modulates calcium and phosphorus homeostasis [7]. Vit D is obtained through the consumption of various foods, but it is mainly synthesized in the skin after sunlight exposure [7]. Vit D adequacy is currently assessed by quantifying serum levels of 25-hydroxyvitamin D [25(OH)D], the circulating metabolite of Vit D [7]. Regarding the respiratory system, Vit D significantly impacts tissue remodeling, reduces inflammatory cytokines and modulates both innate and adaptive immune systems [8]. In COPD patients, adequate Vit D levels have been shown to improve lung function and respiratory symptoms, while also reducing sputum volume and acute exacerbations [9]. Evidence indicates a potential link between OSA and diminished Vit D levels, and CPAP therapy has demonstrated positive effects on Vit D status [10]. OS patients exhibit hypovitaminosis D more frequently compared to OSA patients and healthy controls [11]. However, whether CPAP therapy impacts Vit D status in OS patients has yet to be revealed.

In the present study we aimed to investigate the effect of 12 months of CPAP therapy on Vit D levels in patients with OS and to assess whether compliance with CPAP treatment may have an important role in modifying these levels.

## Materials and methods

### Patients and study design

The enrollment of participants included consecutive patients, who were referred to the sleep laboratory of our institution for symptoms related to sleep-disordered breathing between November 2017 and June 2020. All patients underwent an assessment for COPD and those with concurrent diagnosis of OSA and COPD (OS) were enrolled in the study. All participants gave informed consent prior to enrollment in the study. The study protocol received approval from the Ethics Committee of University General Hospital of Alexandroupolis (IRB: 14 − 1/27.01.2017) according to the Helsinki Declaration of Human Rights.

Individuals who were newly diagnosed with OSA and COPD were included in the study. Inclusion and exclusion criteria have been described elsewhere [11].

Baseline assessments included comprehensive medical history with a focus on the presence of respiratory symptoms, current medication, sleeping habits and tobacco consumption. Age, gender, neck, waist and hip circumference were also recorded, and the waist-to-hip ratio (WHR) as well as the body mass index (BMI) were calculated. For the assessment of daytime sleepiness included patients filled in the Greek version of the Epworth Sleepiness Scale (ESS), a questionnaire that determines the likelihood of falling asleep in a variety of daily circumstances [12]. A score above 10 (> 10) defined the presence of excessive daytime sleepiness [12].

After 12 months of CPAP therapy, assessments of anthropometric parameters and COPD status and symptoms were repeated. Information on COPD exacerbations, medication use and treatment adherence was recorded. Compliance to CPAP therapy was specifically verified using data retrieved directly from the patients’ device.

### OSA and COPD diagnosis and treatment

All participants underwent a Type I sleep study (Alice^®^ 4, Philips Respironics, Murrysville, PA, USA) for OSA diagnosis. Each sleep study was manually scored in accordance with international guidelines [13]. Diagnosis and severity of OSA were set according to the apnea/hypopnea index (AHI) and related symptoms [14].

Individuals included in the study underwent pulmonary function testing (PFTs) (MasterScreen Body, JAEGER^®^, Germany) according to standardized criteria [15]. ]. Arterial blood gas (ABG) analysis was also performed (ABL3000 auto-analyzer, Radiometer Co., Tokyo, Japan) the day preceding polysomnography. COPD was diagnosed and participants were categorized into the “ABE” groups according to the Global Initiative for Chronic Obstructive Lung Disease (GOLD) 2023 guidelines and classification [16, 17].

Following OSA diagnosis, an in-laboratory auto-adjusting CPAP titration study was performed and optimal therapeutic pressure settings were determined according to the AASM guidelines [18]. Good compliance with OSA treatment was defined as an average of CPAP use for at least 4 h per night in at least 70% of nights [18]. All patients received the optimal COPD treatment based on disease severity, as recommended by the GOLD report [17]. All patients received instructions for good compliance to CPAP and were trained in the correct daily use of inhalation devices.

Diagnostic procedures have been described in detail elsewhere [11].

### Vitamin D measurement

Vitamin D levels were measured in serum samples taken upon waking after polysomnography, and again 12 months after the initiation of CPAP therapy. Serum 25(OH)D levels were determined using a commercial radioimmunoassay kit (DiaSorin, Stillwater, MN, USA), in accordance with the manufacturer’s guidelines. Vitamin D deficiency was defined as serum levels ≤ 20 ng/mL.

### Statistical analysis

All analyses were performed using the IBM Statistical Package for Social Sciences version 26.0 (IBM Corp. Released 2019. IBM SPSS Statistics for Windows, Version 26.0. Armonk, NY: IBM Corp). Continuous variables were assessed for normality with the Shapiro–Wilk test. Normally distributed continuous variables are expressed as mean ± standard deviation; non-normal continuous variables are expressed as median (25th -75th percentile). Percentages were compared with the McNemar test and means were compared with the paired t-test. Significance level was set at *p* < 0.05 for all analyses.

## Results

Overall, 46 patients (43 males and 3 females; mean age 60.9 years) were included in the study and evaluated over a 12-month period. The majority (63%) of the participants were former smokers and they were classified in group B, according to the GOLD COPD classification system. Patients’ anthropometric characteristics are reported in Table 1. Polysomnography demonstrated moderate OSA in 13 and severe OSA in 33 patients, with detailed sleep parameters provided in Table 2. Regarding GOLD airflow limitation, most of the patients were in group 2 (FEV_1_ 50–79% predicted). Pulmonary function parameters are shown in Table 3.

**Table 1:** Comparison of anthropometric characteristics of OS patients at baseline and after 12 months of CPAP treatment

	OS patients (*n* = 46)		
	Baseline	12 months	*p*
Gender (males/females)	43/3	43/3	-
Age (years)	60.9 ± 9.1	61.9 ± 9.1	0.998
Neck circumference (cm)	46.7 ± 3.6	45.2 ± 7.6	0.195
Waist circumference (cm)	128 ± 11,9	125.7 ± 17.3	0.234
Hip circumference (cm)	119.3 ± 12.7	120.6 ± 14.3	0.272
WHR	0.96 ± 0.13	1.06 ± 0.15	< 0.001
BMI (kg/m^2^)	37.8 ± 5.5	36.4 ± 8.1	0.128
Tobacco smoking			
Ex-smokers (n/%)	29 (63)	36 (78.3)	0.146
Current smokers (n/%)	17 (37)	10 (21.7)	0.065
COPD GOLD stage (n/%)			
A	14 (30.4)	27 (58.7)	< 0.001
B	20 (43.5)	16 (34.8)	0.180
E	12 (26.1)	3 (6.5)	0.022

Abbreviations: BMI, body mass index; WHR, waist to hip ratio

**Table 2:** Sleep parameters of OS patients at baseline

	OS patients (*n* = 46)
**TST (min)**	315.8 (271.3–350)
**N1 (%)**	12.5 (4.3–20)
**N2 (%)**	72.1 (57.5–84.6)
**N3 (%)**	6.3 (0–15.4)
**REM (%)**	4.9 (0–10.6)
**Sleep efficiency (%)**	84 (72.2–90.5)
**Arousal index**	7 (2–47.6)
**AHI (events/h)**	41.1 (26.1–62.6)
**Aver SaO**_**2**_ **(%)**	91 (87–92)
**Min SaO**_**2**_ **(%)**	74 (64–77)
**T < 90% (%)**	25.8 (9.4–49.1)
**OSA n (%)**	
**Moderate**	13 (28.2)
**Severe**	33 (71.8)
**ESS score**	9 (6–15)

Abbreviations: AHI, apnoea hypopnea index; Aver SaO_2_, average oxyhemoglobin saturation; ESS, Epworth sleepiness scale; Min SaO_2_, minimum oxyhemoglobin saturation; N1, sleep stage 1; N2, sleep stage 2; N3, sleep stage 3; REM, rapid eye movement; TST, total sleep time; T < 90%, time with oxyhemoglobin saturation < 90%

**Table 3:** Pulmonary function parameters of OS patients at baseline and after 12 months of CPAP treatment

	OS patients (*n* = 46)		
	Baseline	12 months	*p*
FEV_1_ (% predicted)	67.8 ± 18.8	72 ± 18.4	0.097
FVC (% predicted)	80 ± 20.6	86.1 ± 21.2	0.018
FEV_1_/FVC (%)	66.3 ± 5	66.4 ± 5.2	0.796
GOLD airflow limitation stage (n/%)			
1	10 (21.8)	15 (32.6)	0.267
2	29 (63)	27 (58.7)	0.424
3	4 (8.7)	3 (6.5)	0.998
4	3 (6.5)	1 (2.2)	0.500
CAT score	9 (6–11)	5 (2–8)	< 0.001

Abbreviations: CAT, COPD assessment test; FEV_1_, forced expiratory volume in first second; FVC, forced vital capacity

After 12 months of treatment with CPAP, significant reductions were observed in: AHI [from 41.1 (26.1–62.6) to 3.1 (1.5–7.6), *p* < 0.001), Epworth sleepiness scale (ESS) score [from 9 (6–15) at baseline to 3 (2–5), *p* < 0.001] and in COPD assessment test (CAT) score [from 9 (6–11) at baseline to 5 (2–8), *p* < 0.001].

At baseline, no association was observed between Vit D serum levels and any of the anthropometric characteristics, PFT results, or polysomnographic measurements. Serum 25(OH)D levels were similar between patients with moderate and severe OSA (20.9 ± 10.2 versus 21.5 ± 7.7 ng/ml respectively, *p* = 0.850).

A subgroup analysis divided patient with OS into 2 groups based on GOLD airflow limitation stages: one group with mild/medium airflow limitation that included 39 patients, and a group with severe/very severe airflow limitation that included 7 patients. Vit D serum levels were similar between groups both prior to (*p* = 0.211) and following (*p* = 0.388) CPAP therapy, and so was the amount of increase of Vit D levels (*p* = 0.455) after 1 year of treatment. Analyzing COPD severity, patients in group A, according to GOLD COPD classification, exhibited higher baseline Vit D serum levels than those in group E (25.8 ± 9.4 ng/ml versus 17.9 ± 7.4 ng/ml respectively, *p* = 0.038). However, this difference between the two groups was attenuated after one year of CPAP therapy (*p* = 0.100) and no difference was noted in the amount of Vit D levels increase between the two groups (*p* = 0.819). Additionally, no significant differences in serum Vit D levels were observed between patients in groups COPD GOLD A and B (*p* = 0.130), or between patients in groups COPD GOLD B and E (*p* = 0.672).

At baseline, no significant association was observed between Vit D serum levels and any of the anthropometric characteristics, PFT results, or sleep parameters. In terms of OSA severity, there was no significant difference in serum 25(OH)D levels between moderate and severe OSA patients (20.9 ± 10.2 ng/ml for mild versus 21.5 ± 7.7 ng/ml for severe OSA patients, *p* = 0.850). Regarding COPD severity, patients in group A, according to GOLD COPD classification, had higher Vit D serum levels compared with those of group E (25.8 ± 9.4 ng/ml versus 17.9 ± 7.4 ng/ml respectively, *p* = 0.038).

**Fig. 1:**
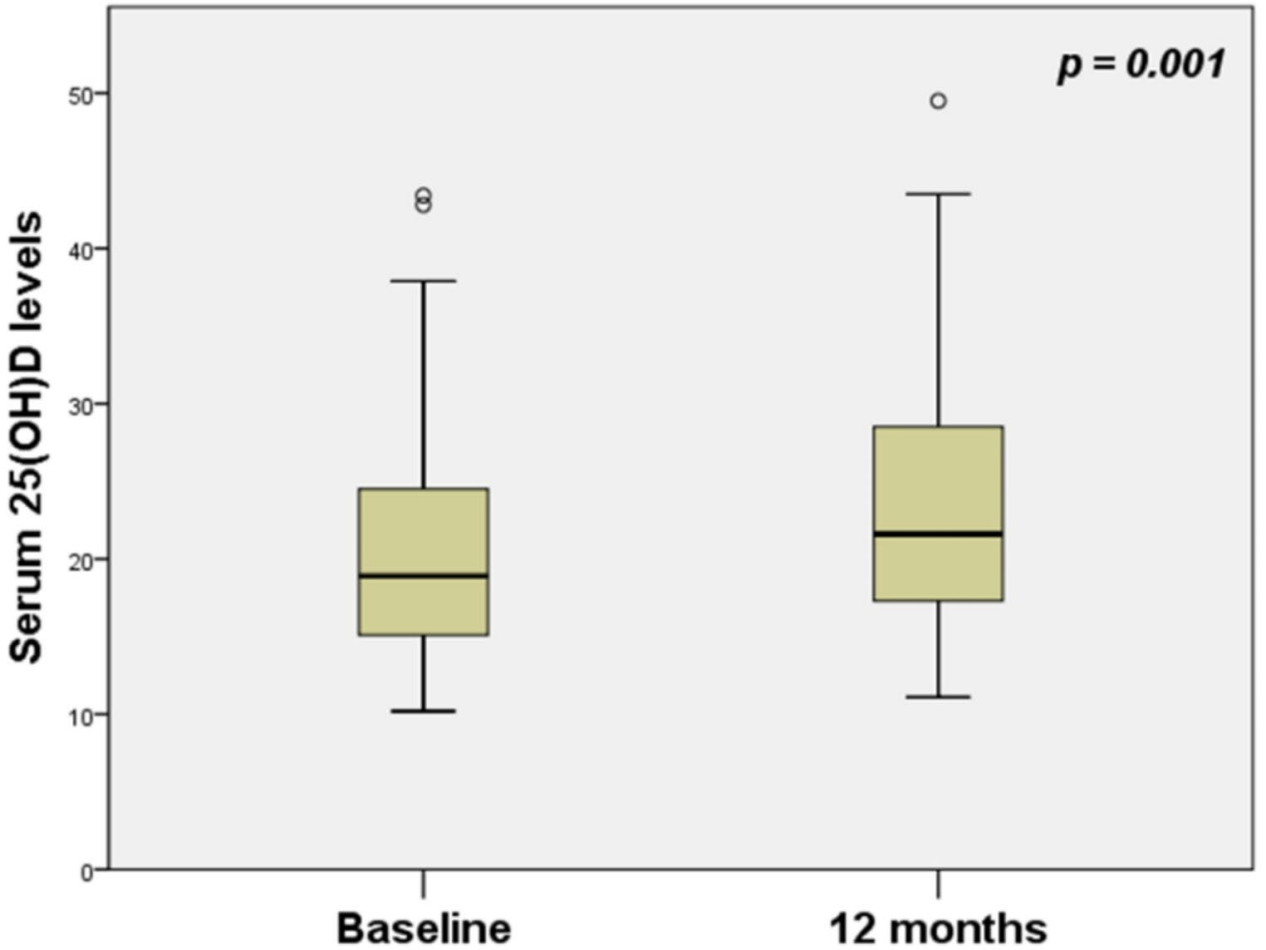
Comparison of 25(OH)D levels at baseline and after 12 months of CPAP treatment

Following 12 months of CPAP therapy, serum 25(OH)D levels increased from a baseline of 21.3 ± 8.4 to 23.8 ± 8.7 ng/ml (*p* = 0.001) (Fig. 1). This increase was observed in patients with both moderate (21 ± 10.2 before versus 24.3 ± 10.6 ng/ml after therapy, *p* = 0.019) and severe OSA (21.5 ± 7.7 before versus 23.6 ± 7.5 ng/ml after therapy, *p* = 0.013).

**Fig. 2:**
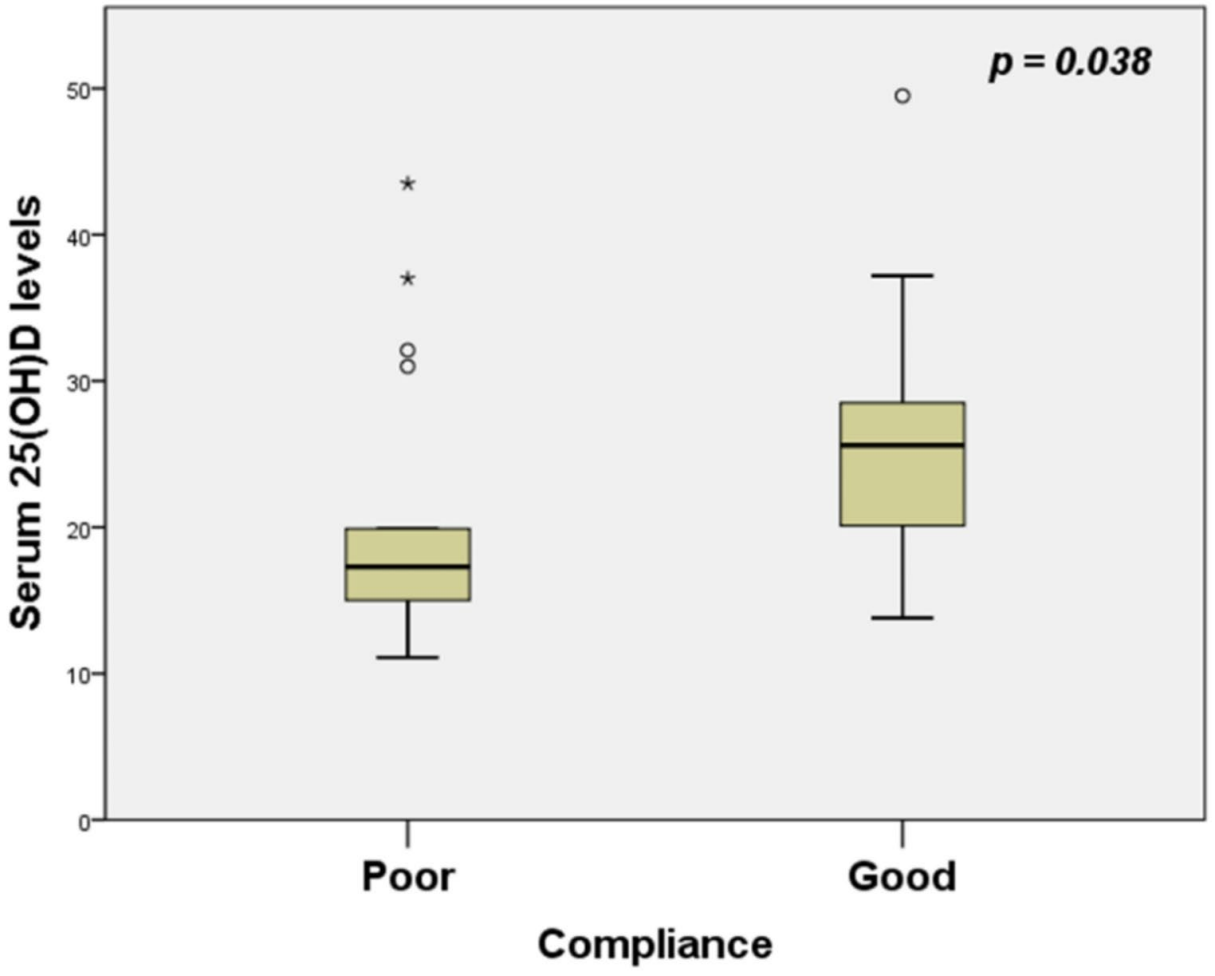
Comparison of 25(OH)D serum levels between OS patients with poor and good CPAP compliance after 12 months of CPAP treatment

Of the participants, 27 were with good- and 19 with poor compliance with CPAP treatment. The first group displayed increased 25(OH)D serum levels compared to the second, i.e., poor compliance (20.4 ± 9.6 versus 25.8 ± 7.6 ng/ml respectively, *p* = 0.038) (Fig. 2). Specifically, patients with good CPAP compliance had a greater increase in Vit D serum levels compared to those non-compliant with treatment (3.4 ± 4 before versus 0.9 ± 3.3 ng/ml after CPAP therapy). Additionally, a greater proportion of patients adherent with CPAP therapy shifted from a deficient to a sufficient Vit D status compared to those with poor compliance with treatment (8 with good versus 0 with poor CPAP compliance, *p* = 0.017). After 12 months of treatment, patients in group A, according to GOLD COPD classification, had higher 25(OH)D serum levels compared to those of patients in group E (26 ± 8.2 versus 14.2 ± 2.5 ng/ml respectively, *p* = 0.001) and similarly patients in group B presented increased 25(OH)D serum levels compared to those of patients in group E (21.8 ± 9.3 versus 14.2 ± 2.5 ng/ml respectively, *p* = 0.048).

## Discussion

Herein, we evaluated serum Vit D levels in OS patients over a period of 12 months of CPAP treatment. Our results indicated an improvement in Vit D levels following CPAP therapy. Additionally, patients adherent to CPAP therapy had higher serum Vit D levels compared to those non-adherent to treatment.

Although a connection between Vit D insufficiency and COPD has been previously documented, the causal nature of this relationship and the potential effects of Vit D supplementation still need to be established [19]. Results from a meta-analysis of 21 studies revealed decreased Vit D levels in COPD patients compared with controls, especially in those with severe COPD and those experiencing exacerbations [20]. In the same meta-analysis, patients with lower Vit D levels were more likely to have COPD and severe COPD, while COPD exacerbations were independent of Vit D status [20]. Furthermore, Vit D supplementation in COPD patients improved lung function, 6-minute walk distance and reduced acute exacerbations, as well as sputum volume and CAT score [9].

Though conflicting, overall evidence suggests that serum Vit D levels are reduced in OSA patients [21, 22]. In a large prospective cohort study, patients with Vit D insufficiency had an increased propensity of developing OSA. This was more evident for obese patients with large waist circumference [23]. Several studies have also identified an association between Vit D status and OSA severity [21, 22, 24]. To further strengthen this hypothesis, in a recent meta-analysis patients with moderate and severe OSA had significantly decreased 25(OH)D levels and presented with Vit D insufficiency more frequently in comparison with non-apneic individuals, regardless of age, BMI and place of residence [25].

Few studies have explored the link between Vit D insufficiency and OS. The 2005–2008 National Health and Nutrition Examination Survey reported a high prevalence of Vit D insufficiency and deficiency among OS patients [26]. We have previously compared 25(OH)D serum levels between 30 OS patients, 30 OSA patients and 30 healthy controls [11]. Patients with OS had diminished serum 25(OH)D levels compared with OSA patients and controls (14.5 versus 18.6 versus 21.6 ng/mL, *p* < 0.001). In the OS group, AHI and FEV_1_ were identified as the principal determinants of serum 25(OH)D levels [11]. At present, there is a paucity of studies examining the effect of CPAP therapy on Vit D status in OS patients.

In the present study, CPAP therapy resulted in an increase of serum Vit D levels in OS patients at 12 months compared to baseline levels. Yet, the effect of CPAP treatment on Vit D levels in patients with OSA remains undefined [27]. Data from a recent meta-analysis failed to corroborate the positive impact of CPAP therapy on Vit D status in OSA patients, and this was regardless of treatment duration [25]. However, the findings of this study should be interpreted cautiously, given the limited number of studies analyzed in the meta-analysis and the inclusion of OSA patients with poor adherence to CPAP therapy. In the study of Liquori et al. [28], 12 months of CPAP therapy resulted in increased serum Vit D levels, with a notably stronger effect among obese patients and those with good adherence to therapy. Transitioning from inadequate to adequate Vit D levels was more common in OSA patients with good CPAP compliance compared to those not adequately using CPAP treatment. Their results were replicated in our study, where adequate treatment adherence was a significant factor for achieving adequate Vit D levels in OS patients and led more frequently to the normalization of Vit D status. Similarly, in the study of Siachpazidou et al., adequate Vit D status was correlated with higher CPAP use both after 3 and 12 months of therapy, with daily CPAP usage emerging as a significant predictor of 25(OH)D levels after 1 year of treatment [29]. In a recent study involving 60 patients, 12 months of CPAP treatment improved bone mineral density and Vit D serum levels in males with severe OSA [30].

In COPD patients, Vit D deficiency may be due to reduced sun exposure as a result of limited outdoor activity, reduced cutaneous synthesis caused by aging and smoking, increased Vit D catabolism resulting from glucocorticoid treatment and lower Vit D storage capacity [31]. Excessive daytime sleepiness, a key feature in OSA, is thought to reduce outdoor activities of OSA patients, limiting sun exposure and reducing cutaneous synthesis of Vit D [32]. Short sleep duration secondary to sleep fragmentation, another main feature of OSA, increases the risk for Vit D insufficiency, and in OSA patients Vit D serum levels have been inversely associated with sleep stage transitions, an indicator of sleep fragmentation [21, 33]. In addition, nocturnal intermittent hypoxia accompanying apneic events negatively affects Vit D status through a mechanism that involves hypoxia-inducible factor (HIF)-1α [34]. Based on the above assumptions, smoking, frequent COPD exacerbations that require glucocorticoid treatment, excessive daytime sleepiness and severe OSA, associated with sleep fragmentation and nocturnal hypoxia could be considered as the main predictors of low Vit D levels in OS patients.

The underlying pathophysiological mechanisms responsible for the positive effects of CPAP treatment on Vit D levels are multifactorial. Data show that OSA patients with short sleep duration are exposed to an increased risk of Vit D deficiency [35]. This is because excessive daytime sleepiness, a common feature of OSA, may limit outdoor activity and reduce Vit D synthesis. Of note, several lines of evidence report that CPAP improves long-term sleep duration, sleep quality and architecture in those patients [36]. Therefore, CPAP therapy by normalizing sleep architecture and stabilizing sleep stage transition, significantly improves daytime sleepiness, decreases fatigue and increases energy in patients with OSA, thus increasing outdoor activity, sun exposure and Vit D skin production [37, 38]. Finally, nocturnal intermittent hypoxia, which is related to apneic events, has been previously associated with low Vit D levels [21]. This is also true for treatment with CPAP, which alleviates nocturnal hypoxia and attenuates its effect on Vit D levels [39].

Nonetheless, our study has limitations. First, the number of patients included in the study was relatively small, but this was due to the small prevalence of OS in the general population. Second, females are significantly under-represented in our study population. This aligns with the low prevalence of both COPD and OS among females. Nevertheless, this gender imbalance could have an impact on the generalizability of the study findings. Lastly, the potential effect of sun exposure and/ or dietary habits among participants was not evaluated. However, all participants reside in the same region of Greece (having similar exposure to sun) and share similar dietary and clothing characteristics.

In conclusion, this study reports that 12 months of CPAP treatment resulted in improved Vit D serum levels in OS patients. This might be due to the improvement of OSA and its related symptoms. The present study highlights the importance of good compliance with CPAP therapy for OS patients, in order to limit sequelae inherent to both OSA and COPD. Further studies with larger sample sizes in this field are still needed.

## Data Availability

Data will be made available on reasonable request.
